# Barriers to accessing health care for people with chronic conditions: a qualitative interview study

**DOI:** 10.1186/s12913-022-08426-z

**Published:** 2022-08-14

**Authors:** Tanja Schwarz, Andrea E. Schmidt, Julia Bobek, Joy Ladurner

**Affiliations:** 1 Austrian National Public Health Institute, Addiction Competence Centre, Stubenring 6, 1010 Vienna, Austria; 2 Austrian National Public Health Institute, Competence Centre on Climate and Health, Stubenring 6, 1010 Vienna, Austria; 3 Austrian National Public Health Institute, Health Economics and Health Systems Analysis, Stubenring 6, 1010 Vienna, Austria; 4 Austrian National Public Health Institute, Psychosocial Health, Stubenring 6, 1010 Vienna, Austria

**Keywords:** Healthcare access, Barriers, Chronic conditions, Multimorbidity, Integrated care, Asthma in children, Chronic back pain, Mental illness, Older people, Austria

## Abstract

**Background:**

There is a growing interest in redesigning healthcare systems to increase access to and coordination across care settings for people with chronic conditions. We aim to gain a better understanding of the barriers faced by (1) children with chronic bronchial asthma, (2) adults with non-specific chronic back pain, and (3) older people with pre-existing mental illness/es in Austria’s fragmented social health insurance system.

**Methods:**

Using a qualitative design, we conducted semi-structured interviews face-to-face and by telephone with health service providers, researchers, experts by experience (persons with lived/ personal experience, i.e., service users, patient advocates or family members/carers), and employees in public health administration between July and October 2019. The analysis and interpretation of data were guided by Levesque’s model of access, a conceptual framework used to evaluate access broadly according to different dimensions of accessibility to care: approachability, acceptability, availability and accommodation, affordability, and appropriateness.

**Results:**

The findings from the 25 expert interviews were organised within Levesque’s conceptual framework. They highlight a lack of coordination and defined patient pathways, particularly at the onset of the condition, when seeking a diagnosis, and throughout the care process. On the supply side, patterns of poor patient-provider communication, lack of a holistic therapeutic approach, an urban-rural divide, strict separation between social care and the healthcare system and limited consultation time were among the barriers identified. On the demand side, patients’ ability to perceive a need and to subsequently seek and reach healthcare services was an important barrier, closely linked to a patient’s socio-economic status, health literacy and ability to pay.

**Conclusions:**

While studies on unmet needs suggest a very low level of barriers to accessing health care in the Austrian context, our study highlights potential ‘invisible’ barriers. Barriers to healthcare access are of concern for patients with chronic conditions, underlining existing findings about the need to improve health services according to patients’ specific needs. Research on how to structure timely and integrated care independent of social and economic resources, continuity of care, and significant improvements in patient-centred communication and coordination of care would be paramount.

**Supplementary Information:**

The online version contains supplementary material available at 10.1186/s12913-022-08426-z.

## Background

Barriers to accessing health and social care represent a key factor in causing health disparities. Regardless of their age, people with chronic conditions often face multiple and complex challenges when trying to identify the right patient pathways already at the onset of their illness or when the first signs of chronic disease appear [1]. People with multiple long-term conditions have also been found to experience worse hospital care than all other patient groups [2]. Further, an international study found that respondents with high morbidity scores report less positive experience with coordination of care compared to those with low morbidity scores, especially among patients with chronic lung, and mental health problems [3]. According to the WHO [4] “developing more integrated people-centred care systems has the potential to generate significant benefits to the health and health care of all people, including improved access to care, improved health and clinical outcomes, better health literacy and self-care, increased satisfaction with care, improved job satisfaction for health workers, improved efficiency of services, and reduced overall costs”. While the WHO’s framework on integrated, people-centred health services is highly valuable, existing frameworks on access to health care as well as studies evaluating integrated patient pathways often focus on care events as the basic unit of interest rather than on patient-centred integrated care across different stages of a patient’s illness [4]. In other words, the integrated care discussion focuses on events rather than on the overall process from a patient’s perspective. Similarly, the WHO’s universal coverage framework focuses on what services are covered, aspects of cost sharing, and population coverage but does not capture inequalities in access at different care stages and/or in settings that are particularly relevant for patients with one or more chronic diseases [5].

As a social health insurance (SHI) system, one of the key challenges in the organisation of the Austrian healthcare system is the fragmentation of organisational and financial structures [6–9]. This applies particularly for people with multimorbidities and/or chronic conditions (ibid.), even if recent reforms have attempted to tackle fragmentation and to shift service provision away from the inpatient sector while expanding outpatient care in the context of target-based governance reforms [7, 10]. In this context, the main stakeholders financing the system (nine provinces, federal level, SHI fund) agreed to continue addressing barriers to access in the reform period 2017–2021 (Table 1) [18].

**Table 1:** Policy context: Austria

Austria is a developed welfare state with an SHI system covering around 99% of the population. The provision of health services in Austria is characterised by relatively unrestricted access to all levels of care including GPs, specialists, and hospitals, and there is no formal gatekeeping system in place [10]. However, in practice, the density of specialists with an SHI contract is low in some rural areas [11, 12], and the proportion of private providers, i.e., without an SHI contract, is rising. In fact, out-of-pocket payments at the point of service are higher in Austria than in other countries with similar levels of health expenditure (17.7% of health expenditure in 2019) [13, 14]. The proportion of people with voluntary health insurance (VHI) is growing [11, 12]. The latter also enjoy shorter waiting times for elective surgery [15] than patients without VHI. Despite high coverage with SHI and being continuously among the countries with the lowest levels of unmet needs in Europe, some authors have reported that a greater proportion of Austrian households are faced with health spending that exceeds their ability to pay than in most high-income countries in the EU [16]. While overall life expectancy is above the EU average (81.7 years in 2017) and rising, healthy life years at birth are substantially below the EU average, with approximately 57 years free of disability at birth versus 64 years in the EU as a whole [11, 12]. Regional variation is substantial, with shorter healthy life expectancy among people living in eastern parts of Austria compared to western parts [17].	

Fragmentation is a major barrier identified in studies on people with chronic conditions in Austria: Patients with chronic conditions face difficulties identifying the right patient pathways, especially when in vulnerable situations, such as persons affected by homelessness [19, 20]. People in lower socio-economic groups are not only at higher risk of being affected by chronic pain symptoms but also have more difficulties dealing with chronic symptoms due to lower health literacy [21, 22]. Socio-cultural factors also contribute to difficulties managing chronic illness [23–26]. Most studies have focused on specific groups, such as people affected by poverty or homelessness, while studies on chronic disease among children or in the general working age population are much rarer. Regarding mental health care or psychosocial care, the main barriers mentioned in the literature range from stigmatisation, information deficits, and waiting times among people with mental health problems (cf. [27]) to barriers to accessing psychotherapy as well as psychiatric inpatient care and psychiatric care in hospital outpatient departments [20, 27–36]. Previous studies have identified financial barriers to accessing psychotherapy, often in combination with other barriers such as long-term unemployment, risk of social exclusion, or language barriers [29–32]. A main barrier includes the ability to navigate a fragmented system, with patients with mental health problems often facing a lack of coordination between care settings and care providers [20, 30, 37]. The vast majority of studies investigating the accessibility of mental health care, however, focused on the working age population (or explicitly on dementia care) as well as on children and adolescents, while studies relating to chronic mental health conditions for the older population (except for long-term care and dementia) are rare (see, e.g., [38] on older people with a substance use disorder in Vienna).

So far, few studies have systematically analysed barriers to accessing health care in the Austrian healthcare system for people with chronic conditions. This paper aims to fill this gap by highlighting such barriers at different stages of a patient’s illness in an SHI country setting in Europe. The study aims to identify barriers to access, with a special focus on patient pathways, in the Austrian context, using three case studies of people with chronic conditions and applying the barrier to access model developed by Levesque et al. [39] as a conceptual framework. The research questions in this study are: What challenges in access do patients with chronic conditions face in the Austrian SHI system at different stages of their illness? How may barriers to access along a patient’s different stages of illness be conceptualised using Austria as an example of social health insurance countries?

## Methods

### Context and conceptual framework

This qualitative interview-based study was commissioned in the course of the Health Reform agreement according to Article 15a of the Federal Constitution on target-based health governance and the agreement according to Article 15a on the organisation and financing of the health care system. A systematic literature review (forthcoming by the authors) served to identify relevant empirical work related to access barriers to health care services in Austria. Based on the review, ten topics were identified for further analysis. The suggested topics aimed to fill gaps in the literature and to cover a broad spectrum of potential access barriers, areas of care, conditions and population groups affected. A national expert group, which is also in charge of driving the healthcare reform, assessed the suggested topics and chose three for further case study analyses. The expert group consisted of representatives of the Federal State of Austria (Ministry of Health), the nine Austrian provinces, and SHI institutions. The case studies were chosen to reflect populations with chronic conditions that are particularly susceptible to inequalities and inequity of access to healthcare services and which were previously under-researched. All case studies followed the same methodology and interview guidelines.

1. **Bronchial asthma in children:** Bronchial asthma is a chronic inflammatory disease characterised by heterogeneous disease patterns due to different environmental, genetic, and economic risk factors. In Austria, it affects about one in ten children [40].
2. **Non-specific chronic low back pain (LBP) in the working population:** Non-specific chronic LBP is a musculoskeletal condition characterised by symptoms in the lumbar area lasting more than 3 months without a diagnosable underlying pathology. According to the Austrian Health Interview Survey (ATHIS), around 25% of the working population suffered from non-specific chronic LBP in the 12 months running up to their participation in the study [41].
3. **Older people with pre-existing mental illness (excluding dementia) living independently:** The most common mental illnesses in old age (> 65 years) include affective disorders such as depression, bipolar illnesses, persistent dementia, acute states of confusion known as delirium, or delusional and schizophrenic disorders [42, 43]. The prevalence of mental illness among persons older than 65 years, excluding dementia, is situated at about 20% according to international studies [43, 44]. Disease patterns and morbidity often change with age [45, 46], especially somatic co-morbidities becoming increasingly relevant, increasing the risk of early mortality [47]. The present study focuses on persons > 65 years with pre-existing mental illness, as these are considered an increasing group as well as being underrepresented in the literature. Dementia was excluded for two reasons, firstly due to the considerable number of ongoing activities in Austria (high awareness for the condition and persons affected) and secondly due to the study concentrating on persons with serious or severe mental illness [47]. Persons living in institutions or with their family, e.g. their parents, were excluded, as they often face different challenges and barriers than individuals living independently.

**Fig. 1:**
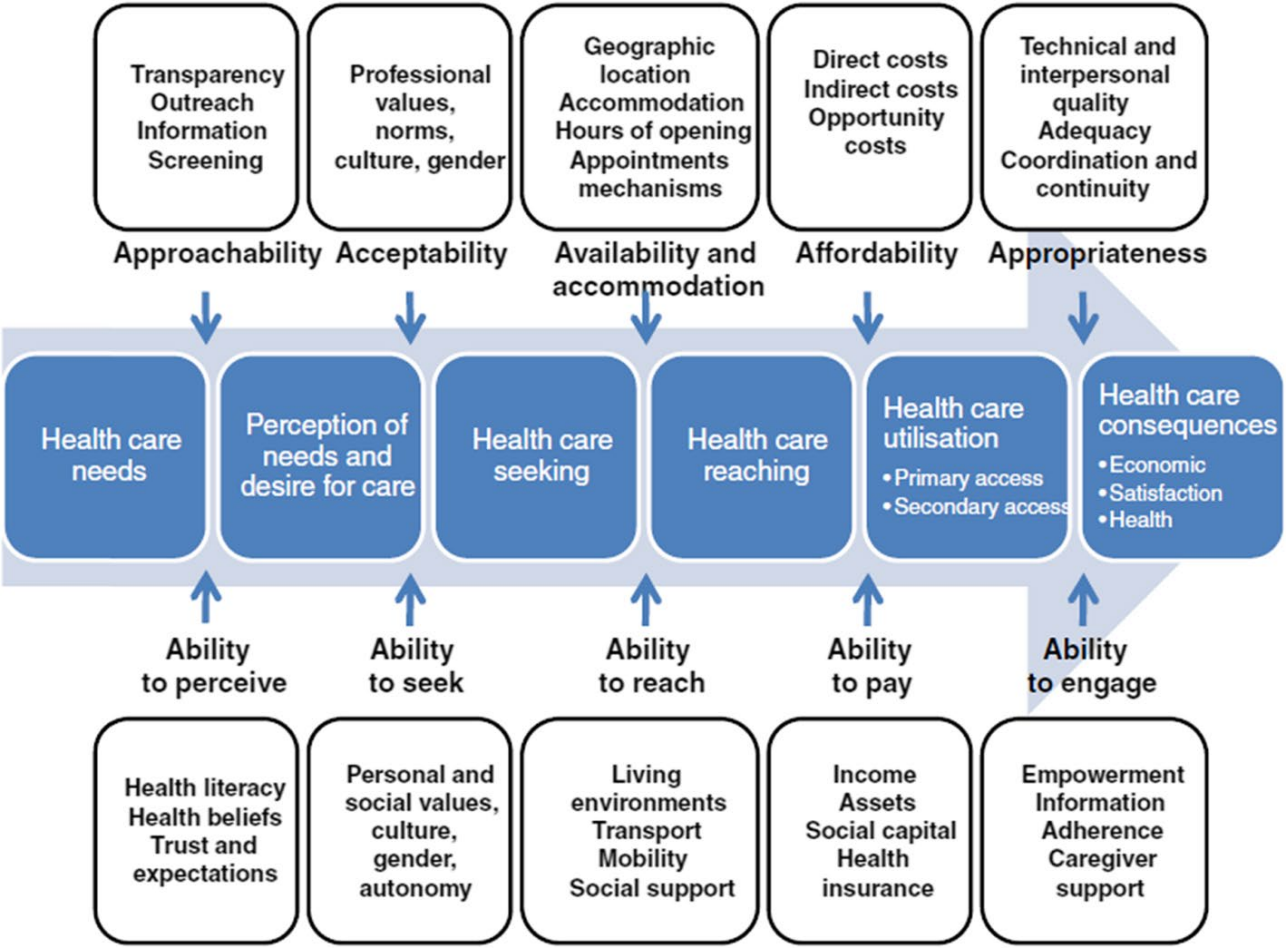
Conceptual framework of access to health care by Levesque et al. [39]. Adopted from Levesque et al. [39]. Permission to use this figure was obtained from Jean-Frederic Levesque

We employed the theoretical framework by Levesque et al. [39] (cf. Fig. 1), who conceptualise access to health care across five dimensions (i.e., approachability, acceptability, availability and accommodation, affordability, and appropriateness), along with five corresponding abilities of populations (ability to perceive, ability to seek, ability to reach, ability to pay, and ability to engage).

1. Barriers to perceiving need (approachability): Perceiving the need for care is often the first step in a patient’s journey and is determined by their individual knowledge and skills (including basic health literacy) as well as any existing (cultural) beliefs about health and illness. The approachability of health services refers to the provider’s efforts to make their services more known to their patients and is often related to transparency, outreach, provision of information and education about services to current clientele [39].
2. Barriers to seeking care (acceptability): Seeking health care relates to cultural and social factors affecting services. It examines whether people will accept services that do not conflict with their personal, social, or cultural values so that they can access them without feeling unsafe or uncomfortable. The ability to seek health or social care also relates to the concept of personal autonomy and the capacity to choose to seek care [39]. Clearly in the case of patients with chronic conditions, some will be able to seek care on their own, while others may need an advocate, perhaps a family member or a professional (social) care worker.
3. Barriers to reaching health care (availability): In this dimension patients’ ability to physically access services is included, based on factors such as personal mobility (e.g., the presence of disability, access to transportation) and external circumstances (e.g., occupational flexibility). In the context of chronic care, the availability of services also refers to the general presence of health services and providers needed as well as their accessibility to patients, both physically and in a timely manner [39].
4. Barriers to utilising health care and barriers to ability to pay (affordability): According to Levesque et al. [39], affordability refers to patients’ economic capacity to spend resources and time in order to use the required services (e.g., direct costs, secondary costs (for getting to an appointment), and opportunity costs (for going on sick leave from work). The ability to pay for health care refers to patients’ economic capacity to pay for actual healthcare services. In the context of patients with chronic conditions, who usually show higher healthcare utilisation rates [48, 49], utilising care particularly includes co-payments and waiting times.
5. Barriers to ability to engage (appropriateness): Health outcomes depend on the appropriateness of care and patients’ ability to engage in health care. Appropriateness of care is reflected in the fit between treatment and patients’ needs, its timeliness and coordination, and its quality. The patients’ ability to engage in health care relates to the their participation in decision making and their compliance in therapy [39]. In the context of chronic care, the appropriateness of care relates to coordination, continuity of care, and interdisciplinary cooperation.

This qualitative interview-based study was conducted and reported in accordance with the Standards for Reporting Qualitative Research (SRQR) [50].

### Participants and setting

The study was conducted in Austria. Eligible interview partners were identified for each case study following a heterogeneous purposive sampling strategy. Purposive sampling method identifies participants with relevant experience and ensures that those who have sufficient knowledge in research scope are selected. Interview partners covered the following four perspectives: health service providers (in and outpatient services), experts by experience (persons with lived/personal experience i.e., service users concerning one of the three conditions, patient advocates or, family members/carers), research, and public health administration. The research team contacted interested participants to schedule a face-to-face or telephone interview. Diversity in age, gender and residence (urban or regional/rural) was ensured where possible. Recruitment continued until theoretical saturation (i.e., no new emerging themes) was reached within each population group with chronic conditions. All participants provided written and verbal consent.

### Data collection

Qualitative data were collected through expert interviews [51]. A semi-structured interview guide (available as supplementary material) was developed through literature review and consulting experts and followed the dimensions of barriers to access from Levesque et al. [39]. The interviews were conducted by experienced qualitative researchers in accordance with the predetermined interview guide. TS conducted the interviews related to bronchial asthma in children, JB those on non-specific chronic low back pain in the working population and JL those on older people with pre-existing mental illness (excluding dementia) living independently, according to the researcher’s expertise. In total, 25 interviews were conducted between July and October 2019. Participants were only asked about those conditions for which they had sufficient expertise, i.e. (A) bronchial asthma; (B) non-specific chronic lower back pain or (C) mental health (see Table 2). All interviews were audio-recorded and subsequently transcribed and took 40 to 90 minutes; three were conducted by telephone and the others face-to-face in hospitals, treatment facilities, at the authors’ research facility and at participant’s homes. After the introduction of the study and its objectives, key questions were asked with a focus on the following four areas: (1) identification of barriers to healthcare access, (2) influencing factors and (3) possible reasons for barriers mentioned, and (4) measures and recommendations for overcoming or reducing these barriers (see supplementary material). These four areas were chosen to present a broad view of the actual situations of people with chronic conditions and enabled us to adopt a holistic perspective. During the interview, follow-up questions (i.e., further explanations or examples) were offered to draw out additional information when necessary. To close the interview, the participants were asked if they had anything else, they would like to share.

**Table 2:** Overview of interview sampling

Interview	Gender	Perspective	Type of care provided (where applicable)	Region	(Medical) expertise (where applicable)	Setting (where applicable)
1A	F	research	–	Vienna	general medicine	urban
2A	M	provider	inpatient	Vienna	paediatric pulmonology and allergology	urban
3A	M	provider	inpatient	Salzburg	paediatrics	rural
4A	M	provider	inpatient, rehabilitation services	Styria	paediatrics	rural
5A	F	expert by experience		Austria	carer, patient advocate	national
6A	M	expert by experience		Austria	patient advocate	national
7A	M	expert by experience		Austria	patient advocate	national
8A	M	expert by experience		Austria	patient advocate	national
9B	M	research	–	Styria	–	national
10B	F	provider	outpatient	Vienna	clinical psychology	urban
11B	F	provider	outpatient (rehabilitation)	Vienna	physical medicine and rehabilitation	urban
12B	M	provider	inpatient	Upper Austria	neurology	national
13B	M	provider	inpatient	Carinthia	anaesthesia and intensive care medicine	national
14B	F	public health administration	–	Styria	–	urban and rural
14B	F	expert by experience		Austria	patient advocate	urban
15C	M	research	university, consulting	Vienna	psychiatry	(inter)national
16C	F	provider	outpatient	Vienna, Austria	general medicine	urban and rural
17C	F	provider	outpatient (home treatment)	Vorarlberg	nursing	urban and rural
18C	M	provider	outpatient	Vienna	psychiatry	urban
19C	M	provider	outpatient	Styria	psychiatry	urban
20C	M	provider	inpatient	Styria	geronto- psychiatry	urban
21C	F	expert by experience		Vienna	service user	urban
22C	F	expert by experience		Upper Austria	service user	rural
23C	F	expert by experience		Vienna	carer	urban
24C	F	expert by experience		Vienna	carer	urban

A: bronchial asthma; B: non-specific chronic lower back pain; C: mental illness

Participants were only asked about those conditions for which they had expertise, i.e. A bronchial asthma; B non-specific chronic lower back pain or C mental health

### Data processing and analysis

All interviews were audio-recorded and transcribed verbatim by the researcher who conducted the interview concerned. Transcripts were not returned to the participants for comments. The research team (AS, TS, JB, JL) applied thematic analysis by using the “framework analysis approach” to manually analyze the interview transcripts [52]. Over-arching themes and sub-themes related to barriers to healthcare access were developed in accordance with the theoretical framework by Levesque et al. [39] to summarise the data. Data were thematically summarized and placed in the appropriate dimension of the theoretical framework [39], also the themes and sub-themes were compared with each other. Differences in researcher interpretation of the data were resolved through discussion. Subgroup analysis was conducted on participants’ data depending on their professional role and work setting. For the final step of the framework analysis, findings were discussed, and interpretations agreed between the researchers.

## Results

The findings are organised along the dimensions from Levesque et al. [39] and summarised for each case study: (1) bronchial asthma in children, (2) non-specific chronic LBP in the working population, and (3) older people with pre-existing mental illness/es (excluding dementia). In addition, Table 3 provides a summary of barriers to access by dimension, taking the different perspectives into account, namely whether barriers refer to demand side (the patient’s perspective) or supply side factors (the provider’s perspective). It represents a synthesis of the results from a cross-thematic perspective and as such an important element for the overall findings of our study. While we did not differentiate supply and demand side factors within each condition below, we highlight this distinction in Table 3 as well based on the original framework by Levesque et al. [39].

**Table 3:** Summary of barriers to access by dimension

	Factors primarily influencing the demand side	Factors primarily influencing the supply side
**Barriers to perceiving need (approachability)**	» **Low or limited health literacy** affects patients’ perceptions of health needs, their ability to navigate the health system and follow appropriate treatment pathways as well as to accept and follow indicated therapy.	» **Patterns of poor patient-provider communication** or **ineffective patient-centred care** restrict the approachability of health services and information exchange, often leading to reduced quality of care. » **Lack of awareness** of the patients’ social and family background prevents providers from identifying individual help-seeking behaviour and specific treatment needs.
**Barriers to seeking care (acceptability)**	» **Fear of stigmatisation** is prevalent and often accompanied by a rejection of the psychological component of the disease, resulting in individuals potentially avoiding and/or delaying seeking professional help for mental health problems. » **Limited consultation time** constrains patient’s care-seeking behaviour and acceptability of services.	» **Insufficient consideration** of comprehensive therapy approaches including psychological and social treatments is a major hurdle to successful treatment.
**Barriers to reaching health care (availability)**	» **Previous experiences of treatment** can have a significant influence on the behaviour of those seeking help.	» **Structural barriers** such as a lack of infrastructure, inadequate transportation, or waiting times affect families and patients particularly when their socio-economic situation does not allow them to switch to the private sector. » The **urban-rural divide** exacerbates barriers to accessing specialists accompanied by longer distances and travel times to reach (specialist) care. » The **limited (regional) availability of specialists with an SHI contract** results in individuals delaying specialist examinations or consulting primary healthcare providers instead, who often have limited knowledge of or experience with a certain condition and its associated specific needs. » The **strict separation** between social care and the healthcare system highlights the lack of structured cooperation and consistency throughout the system. » **Holistic therapy approaches (bio-psycho-social)** such as multimodal pain therapy in primary care settings or outpatient rehabilitation options are lacking.
**Barriers to utilising care and barriers to ability to pay (affordability)**	» **Co-payments** (e.g., for psychotherapists, physiotherapists, etc.) or private practice consultations, as a result of limited public capacities or waiting times, are a financial burden especially for patients with limited economic resources. » **Waiting times** are long for those patients who cannot afford (to switch to) elective private practice consultations. » **Language skills, level of education and health literacy** can affect the understanding of and adherence to certain treatment measures thus resulting in different health outcomes.	» **Limited consultation times** reflect the lack of representation of doctor-patient time in the SHI reimbursement scheme.
**Barriers to ability to engage (appropriateness)**	» **Inconsistent and uncoordinated care pathways** lead to doctor hopping on the part of patients, self-medication, avoiding seeking care, decreasing trust, and inefficiencies within the care process. » A **lack of continuity in care especially** in transition phases (e.g., paediatric to adult medicine) may lead to gaps in care or reduced adherence to therapy.	» **Missing or insufficient (adherence to) treatment guidelines** lead to incorrect recommendations or even to manifestation of the disease concerned.

### Barriers to perceiving need (approachability)

#### Bronchial asthma in children

The perception of needs is not solely influenced by general age- and development-related factors, but also by socioeconomic factors, including education level, income, and migration background [53, 54]. Despite high standards of health care in Austria, patient organisations pointed out that it can be challenging for parents to find their way around services and obtain advice (5A-8A). Communication issues affect the approachability of health services and the transparent and adequate provision of information to families (1A). Delayed perception of need due to limited health literacy on the part of children or parents was also mentioned as a main concern (1A, 2A, 6A), the importance of *“mutual communication and a good quality of conversation between doctor and patient”*(1A) being emphasised.

#### Non-specific chronic LBP in the working population

Provider interviews revealed that patients with non-specific chronic LBP frequently have poor body perception and/or health literacy. They tend to wait too long before seeking care, risking symptoms getting worse. *“People react too late. They tend to recognize back pain as such too late. In the early stages, they do not see back pain as a serious illness and think it will get better.” (9B).* Providers report experiencing cultural differences relating to how pain is perceived and expressed. The analyses based on ATHIS data confirm these perceived differences in the perception of pain between persons with migration background and without [53].

#### Older people with pre-existing mental illness

In the case of mental illness, all experts stated that a disease-related lack of perception and/or a distorted perception of the patient’s own health status *(“I am fine, I am not ill”)* and their associated health needs *(“I do not need help”)* can be a central barrier; according to one outpatient provider, home visits often reveal “*catastrophic conditions, e.g., open wounds”* (17C). The experts interviewed pointed out that limited health literacy and low awareness of mental and somatic health problems make it difficult for those affected to recognise their need for help. The family background, including behaviour, can also play a crucial role in perceiving a health problem and influences whether help is sought: “*It is not so much about education, but how mental illness has been dealt with in the family (openly or covertly)”* (22C)*.*

### Barriers to seeking care (acceptability)

#### Bronchial asthma in children

Knowledge about possible risk and protective factors such as previous experience of illness or family and cultural background were found to be crucial to improve the acceptance of services and the conduit of information between healthcare providers and children and their families (1A, 3A), especially in the early phase of the care pathway. Providers (2A, 3A), report that it can be difficult for them to reach non-German-speaking parents and to adequately convey to them the importance of therapy (for their children). Further, the success of training measures can be smaller for persons/children of persons with limited language skills/competence and/or health literacy, as these factors pose barriers to good and effective communication [55].

#### Non-specific chronic LBP in the working population

In almost all interviews (providers, patient advocates, public health administration), it became clear that non-acceptance of the psychological component of the disease and, consequently, rejection of comprehensive therapy including psychological and social treatments is a major hurdle to successful treatment. Fear of stigmatisation was mentioned as a possible cause. Providers and the patient advocate also mentioned a lack of information and inadequate doctor-patient communication as barriers to access. Examples included “*Your spine looks terrible*”, “*You are imagining it*”, “*Pull yourself together*” (11B). Interviews also revealed that poor linguistic skills/competence and/or limited ability to express oneself (e.g., when describing health problems, providing information on one’s own medical history, expressing needs, talking about feelings, etc.) can be especially problematic if consultation time is limited.

#### Older people with pre-existing mental illness

Stigma for patients with mental illness significantly affects their help-seeking behaviour [56, 57]. Shame or pride often prevents people from seeking and/or accepting help from health and/or social services. Others avoid “the system” or being associated with it for various reasons (negative experiences, not wanting to declare themselves mentally ill, etc.): “*Someone could see that I am different”,* “*I don’t want to do that to my family, going to a psychiatric hospital”,* or “*What will people think?”* were reasons listed by one provider (17C). Patient advocates, providers, and family members pointed out that previous experiences of treatment can also have a significant impact on the behaviour of those seeking help: “*If the first contact does not go well, I no longer go, I feel excluded”* (21C)*.* It is also perceived to be stressful “*having to explain yourself again and again”* or “*having to explain again and again what you need”* (21C, 22C).

### Barriers to reaching health care (availability)

#### Bronchial asthma in children

According to the interviewees, a clear urban-rural divide with longer distances and travel times as well as a lower number of paediatricians in rural areas represents a barrier to adequate care for asthma patients. Consequently, GPs, who are usually located closer to families’ homes, are consulted more frequently than paediatricians. Experts considered this situation to be problematic as many GPs have rather limited knowledge of asthma and allergies, especially among children, leading to specialist examinations often being carried out too late or the causes of symptoms not being properly recognised. An example for improper treatment mentioned by one patient advocate is that “*asthma attacks in a child have been treated with cough syrup for a long time” (6A)*. According to the interviewees, the issue of limited knowledge also applies to hospitals in rural areas.

As children spend a substantial amount of time at school, the school system is a critical environment for their asthma management. Additionally, the interviewees pointed out that the school setting may offer access to health care to those who may not otherwise use the healthcare system consistently (1A, 5A). However, many experts criticised the current, strict separation between school and healthcare environments (1A, 5A, 8A). The lack of structured cooperation or links between these two areas leads to considerable stress, including unfavourable effects on children’s success at school, but may also result in serious health disadvantages. As many children need constant medication and quick help from adults in an emergency, a collaborative effort between the healthcare system and schools is critical for improving asthma outcomes and reducing asthma-related barriers to learning. “*Teachers must be aware of the importance of their educational actions: Sensitive measures are needed and an open discussion with the class about the health of the child concerned”* (1A) is how one expert put it.

#### Non-specific chronic LBP in the working population

Structural barriers such as poor infrastructure or long waiting times were mentioned most frequently by the experts interviewed. Especially for patients whose socio-economic situation does not allow switching to the private sector, there were long waiting times for accompanying therapy such as physiotherapy or psychotherapy which ranged from 2 weeks for an outpatient practice appointment, 2 months for SHI-contracted psychotherapy, and 3 to 6 months for outpatient pain clinics. As waiting times sometimes prolong the length of patients’ sick leave, one provider highlighted workplace fears (absence from work, losing one’s job) as an additional mental stressor. Another expert (14B) mentioned commuters as an example, *“who come home only for the weekend and have to go back on a job to Vienna on Monday for assembly. They get a painkilling medication and then they’re fine again”.*

#### Older people with pre-existing mental illness

Barriers to seeking and reaching healthcare services might be due to general, age-related limitations but also related to the individual circumstances of a person’s living situation. Factors such as poorer vision and/or hearing, insecurity, dizziness, forgetfulness, or multimorbidity on the one hand and living in rural areas, being confronted with long distances, and limited (public, private) mobility options on the other hand may lead to withdrawing, less confidence, and can deter older people with mental illnesses from seeking and accessing health services.

Providers and affected individuals indicated that social psychiatric services are not well equipped for the needs of older people with mental illness, especially for those with physical comorbidities or special age-related requirements (e.g., with respect to mobility, need for physical assistance/care). The physical needs of this group sometimes may not be addressed sufficiently. According to several statements from experts (providers, relatives), ageism, or a distorted public image of the elderly, could well be a general societal issue. As one provider commented in this context, “*society has great problems approaching the issue of old age”* (20C), adding that barriers increase for older persons with mental illness as discriminating factors (age- and mental-illness related) come together in a cumulative way.

### Barriers to utilising care and barriers to ability to pay (affordability)

#### Bronchial asthma in children

Social factors may affect the possibility to access services. According to the experts interviewed, the lack of paediatricians with a SHI contract may place increased social burdens on patients and caregivers in the context of utilising healthcare services. While, on the one hand, many contracted doctors no longer accept new patients or long waiting times are the norm, the cost of consultations and treatment by elective doctors in private practices can put a high financial burden on socially disadvantaged families; in other words, it is not an affordable option for them.

Ineffective communication between patients and providers was introduced as another hindering factor for the optimal utilisation of needed care by the participants. According to one interviewee, *“it is difficult to achieve the necessary commitment and understanding for asthma training”* (2A), due to the very limited appointment time SHI paediatricians have with their patients. Compliance with and success of certain measures such as inhalation and physiotherapy techniques or hygienic use of specific equipment require a certain degree of understanding (health literacy and language skills). Limited language skills and/or lower level of education can, next to limited consultation time, potentially pose an additional barrier. Care pathways are not clearly defined in Austria, neither generally nor for chronic conditions, resulting in patients accessing the health care system via different routes (e.g., GPs, paediatrician, medical specialists, hospital), thus also experiencing different outcomes e.g., related to diagnosis or case management.

#### Non-specific chronic LBP in the working population

The topic of diagnostic imaging was very prominent in the interviews. Although current guidelines do not recommend diagnostic imaging, a potential oversupply had been observed by the experts (providers, researcher). Reasons for oversupply mentioned were: *“Patients demand diagnostic imaging by using acquaintances who also received diagnostic imaging as their leverage. Doctors who use imaging diagnostics for reasons of legal protection.*” (9B). *“Many patients get imaging every few months. Doctor shopping is an issue here. Patients change doctors because they are not getting better and often get another imaging from that doctor.”* (12B). Experts (providers) viewed this development critically, as diagnostic imaging might lead to wrong diagnostic associations as well as to unfavourable and rigid disease concepts, making patients more likely to adopt passive attitudes and to shift responsibilities to medical personal, which could lead to iatrogenic chronification.

According to providers and patient advocates, detailed counselling interviews are important to understand the complexities (physical, psychological, social) of the disease, but also to build trust between patients and their doctors. However, both providers and patient advocates stressed the limited time frame for a detailed medical consultation, especially in outpatient practices. One explanation suggested by both groups is the current SHI reimbursement scheme, which does not sufficiently reflect the needs of patients with non-specific back pain, namely sufficient doctor-patient time and active rather than passive treatment measures: *“Doctors do not listen enough, they have too little time. They are only reimbursed for 15 minutes of patient consultation. Instead they prescribe medication or refer to psychiatrists”* (14B).”*Doctors sometimes have too little time to manage these patient group properly. A pain assessment takes time, which is not rewarded on behalf of SHI. Instead infiltration is the measure of choice […] If patients do not speak German, the consultation takes even longer.”* (11B).

The patient advocate and the expert working in public health administration mentioned individual co-payments as a barrier, especially when it comes to elective medical or therapeutic services (e.g., physiotherapy or psychotherapy). Patients with limited economic resources are hit hardest by this barrier, with therapy being more complicated or even impossible [13].

#### Older people with pre-existing mental illness

All interviewees reported deficiencies in the provision of care often accompanied by long waiting times and a high financial burden. Some noted that patients are often “forced” to switch to elective doctors in private practices due to long waiting times in the public (outpatient) sector. Those whose financial resources do not allow this are at a disadvantage and face less choice, longer waiting times, and delayed treatment. One expert admitted that “*those who cannot afford to pay are left behind”* (15C) and one affected person commented that “*if you pay for it yourself, you will get it faster”* (22C).

Several providers noted that co- or multimorbidity (i.e., mental and somatic) as well as medication (i.e., changes in medication, their interactions, and polypharmacy) are complex for this patient group. The research expert found that providers often lack knowledge and experience in the field of drug administration but also emphasised that *“evidence is difficult to transfer into practice” (*15C). In some cases, medication may be discontinued too early due to providers’ and/or patients’ lack of knowledge (i.e., effect of antidepressants only after 6–8 weeks).

### Barriers to ability to engage (appropriateness)

#### Bronchial asthma in children

Well-managed outpatient paediatric asthma care generally leads to better asthma control. However, effective strategies to improve childhood asthma outcomes rely on a multidisciplinary, cross-sector approach, with an emphasis on addressing social determinants. From a provider’s point of view, a societal trend reversal can be observed: while in the past parents first consulted paediatricians, nowadays they tend to consult specialised physicians such as pneumologists or allergists directly given that there is no gatekeeping system in place for access to specialist care in Austria.

Patient advocates repeatedly pointed out that there is no standardised concept for the transition of chronically ill young people to adult medicine in Austria and that the quality of further care “*depends purely on whether the paediatrician in charge knows a specialised colleague or is committed to find one”* (5A). The interviewees all agreed that in this phase adequate care is of particular relevance as adherence to therapy often starts to decline from puberty onwards and *“a certain resistance is noticeable among patients”* (3A). In the experience of the patient advocates, *“insufficient compliance at this age often leads to an acute deterioration in the patient’s state of health and serious long-term effects”* (6A).

#### Non-specific chronic LBP in the working population

Insufficient application of current national guidelines was repeatedly mentioned by researchers and providers, with the providers reporting a lack of knowledge about guideline-based treatment (e.g., multimodal pain therapy) in the outpatient sector, in particular among GPs and orthopaedists. According to these experts, ignorance of guidelines is particularly problematic at the onset of a patient’s pain symptoms. Incorrect recommendations (e.g., passive treatments) or even iatrogenic suggestions (e.g., immobilisation) may promote the manifestation of the disease. Further, pain medicine is, according to the interviewed experts, not sufficiently represented in the curricula of medical study programmes and there may not be enough incentive to obtain post-graduate qualifications.

All interviewees addressed a lack of interdisciplinary coordination, with patients seeking several medical opinions, self-medication, and fragmented patient pathways at all levels of care (hospitals, hospital outpatient departments, GPs, and specialists). Information gaps among practitioners compound inefficiencies within the care process as a whole (e.g., repeated imaging).

The interviewees from research and public health administration reported compliance problems in the treatment of chronic non-specific LBP with very time-intensive therapies not always being compatible with personal commitments. According to the public health administration expert, this may be especially problematic for shift workers as well as those with family and private obligations. Equally, several experts (patient advocate, providers) mentioned the cost of elective therapies represent a barrier in this group. Another factor mentioned by providers is a patient’s employment status. For unemployed patients, the lack of daily structures may prevent them from keeping appointments or, the prospect of retiring early may add a secondary gain to being ill (pension payments).

#### Older people with pre-existing mental illness

Shortcomings in coordination, cooperation, and communication between different service providers were stated as a source of frustration by all interviewees and were among the most frequently mentioned barriers to care for older people with mental illness. The importance of continuity of care and trust was emphasised. An expert by experience pointed out that “*it is difficult to gain or build new trust when practitioners change and there is always a new face in front of you”* (21C). On the other hand, selective information provided by patients was mentioned as one reason for inaccurate and discontinuous care. Relatives reported that patients tend to describe symptoms as being less serious in order to avoid potentially necessary inpatient treatment.

Affected individuals had the impression that somatic providers were overwhelmed by the mental symptoms of their patients: their mental symptoms were not thematised, no efforts to coordinate were made by the providers, and there was little dialogue between different providers and with patients. Rather, communication and coordination were delegated to the patients and their relatives for the reason that providers *“want to get rid of them [the mentally ill elderly] as soon as they are more or less stabilised”* (23C). Consequently, a holistic view of a patient’s health status and information on previous consultations with other doctors was fragmented: “*healthcare providers are not informed about others unless the patient reports it”* (24C).

## Discussion

Beyond questions of who is covered, what services are covered, and what proportion of costs is covered, countries with almost universal coverage may still have barriers in their healthcare systems for people with chronic conditions. While studies on unmet needs suggest a very low level of barriers to accessing health care in the Austrian context [13, 58], our study highlights potential ‘invisible’ barriers that go beyond structural factors such as universal coverage. Our study provides insights into existing inequalities and inequity when navigating the healthcare system and identifying the right patient pathways, using the examples of children with bronchial asthma, adults with non-specific chronic LBP, and older people with pre-existing mental illnesses. Its findings suggest that there are gaps and barriers to accessing health services for people with chronic conditions regardless of their age; as a result, they experience considerable challenges when trying to navigate the health system to access the services they need.

The case studies were chosen to reflect populations that are particularly susceptible to inequalities and inequity of access to healthcare services and which were previously under-researched; we thus aimed to cover a broad spectrum of barriers to areas of care for people with chronic illness. While the Levesque et al. [39] framework was helpful in guiding our review of barriers to healthcare services and offered an insightful way of examining the case study populations included in this study, the importance of barriers in coordination among care providers was only partially captured by the framework, although this is particularly relevant for people with chronic conditions.

Our findings indicate the importance of an integrated approach in care, especially for people with chronic conditions. It is well known that people with chronic disease are often affected by more than one illness, which is why moving away from a single-disease framework towards a patient-centred model [59] is recommended, or towards a function-oriented approach focusing on “whether a patient can function in a way that they find acceptable” rather than focusing on single parameters such as blood pressure reduction [60]. The gradual introduction of a primary healthcare model in Austria is a first step towards such an approach as it often comprises multidisciplinary teams, including physicians, nurses, physiotherapists, social workers, and mental health staff [61]. Primary care models are patient-centred, and collaboration between professionals as well as the distribution of their roles change according to patient needs [62]. Further, innovative approaches such as the piloting of so-called social prescribing models in Austria may help patients with chronic conditions to benefit from non-medical referral options that can be delivered alongside existing primary care services [63]. Embedded in the voluntary and community sectors, social prescribing can address complex health, psychological, and social issues presented in primary care, and there is increasing interest in its potential to reduce health inequalities and emergency department service demand [64, 65].

A wide range of social determinants at individual and policy level create and influence access barriers, from the identification of healthcare needs to health consequences. Significant and recurring challenges to access reported by providers and patient advocates alike predominantly focused on patients’ ability to perceive a need and to subsequently seek and reach healthcare services. Across all case study populations, insufficient health literacy was identified by the providers as affecting patients’ perception of health needs and impeding individuals’ ability to navigate the health system. Further, communication issues affect the approachability of health services and adequate information exchange. In the cases highlighted by our study, a number of barriers were found to be unique to, or more prominent among, certain populations. For instance, while fear of stigmatisation and discrimination is highly prevalent among older people with mental illnesses and adults with non-specific chronic LBP, society generally has a non-judgmental and accepting attitude towards patients with bronchial asthma. It was highlighted that a lack of awareness of mental and/or somatic illnesses and insufficient consideration of comprehensive therapy approaches on the part of patients and providers alike often result in individuals avoiding or delaying seeking professional help for mental health problems. These results are in line with previous findings indicating that specific knowledge about treatment improves help-seeking and service use and that gaining knowledge about mental illnesses is one motivation to seek help [66].

Limitations in the ability to seek and reach healthcare services become particularly evident as the demands on patients to orient within and navigate health care systems are increasing [67]. In Austria, the context of this study, the fragmentation of the healthcare system as well as the social sector in relation to organisational and financial matters often poses a challenge and was described as complex, confusing, and difficult to navigate for patients. Such fragmentation across sectors is an additional complicating factor as social issues such as unemployment, low income, allowances, or care needs disproportionately affect people with multimorbidities and/or chronic conditions. Structural barriers such as a lack of infrastructure, inadequate public transport, long waiting times, and the limited (regional) availability of specialists with an SHI contract were frequently cited challenges for chronically ill patients. These findings are similar to those in a relevant study that found structural factors, such as access to transport or financial resources, to be a prerequisite for generating self-management capacity in patients with multimorbidity [68]. Likewise, patients of low socioeconomic status face additional barriers to accessing care [53]. Our findings suggest that system-based barriers disproportionally affect families and patients who experience challenges organising themselves and whose socioeconomic situation does not allow them to compensate for these potential disparities with financial or social resources. More drastically, patients with the greatest support needs, such as those with multimorbidities or chronic conditions, may often be left behind.

The results also reveal certain particularities for our case study populations. With regard to bronchial asthma, children’s health capital is dependent on their parents’ social situation and ability to utilise healthcare services. Still, according to our interviewees, asthma care pathways are usually better defined compared to non-specific chronic LBP or mental illnesses. Strict separation between school and other social settings and the healthcare system as well as disrupted continuity of care, especially in the transition phase from paediatric to adult medicine, were found to pose specific barriers for children’s health outcomes, however. The issue of transitioning asthma care from adolescents to adults is of major importance as the risk of respiratory morbidity increases in adulthood [69]. A recent review also found that once patients are transferred to adult medicine, limited knowledge of their condition, limited understanding of how to manage related symptoms and comorbidities, and limited comprehension of medical indications often persist [70]. Similarly, the burden of non-specific chronic LBP has implications for both ageing employees and employers. Many obstacles are faced by people with chronic pain when looking for employment or returning to work after a period of absence [71–73]. If the cause of back pain is associated with workplace factors, a return to an unchanged workplace may not be successful and recovery may be impeded, regardless of appropriate treatment for the disorder [74].

Our findings underline the role of necessary structures and conditions of health and social care systems. By examining the results along the dimensions based on Levesque et al.’s [39] framework, we found that barriers involving coordination among care providers are strongly interlinked and that patient-centred communication is key if health outcomes are to be improved. Further, shortcomings in coordination, cooperation, and communication at the very beginning of patients’ treatment pathways are likely to impair the course of future treatment, leading to an exacerbation of existing barriers. The close interactions between poor communication, uncoordinated care pathways, and, ultimately, poorer health outcomes become particularly visible when addressing chronic conditions, confirming the importance of a holistic and patient-centred approach.

A particular strength of this study was the inclusion of four different perspectives. The heterogeneous sample consisting of health service providers, experts by experience (persons with one of the three conditions, their advocates or carers), researchers, and public health administration enabled a comprehensive, multidisciplinary assessment that gives an indication of the accessibility of the healthcare system in Austria for people with chronic conditions as well as a broader view of the changes required by stakeholders in the future. While we have captured some patients’ perspectives by proxy through provider interviews, we acknowledge that this gap may have significantly limited our insights into their unique experiences of access. Nevertheless, our interview partners demonstrated a high level of agreement concerning the key issues for people with chronic conditions. Although this article only concerns barriers to accessing the Austrian healthcare system, the results may also be useful in countries with similarly functioning systems, that is decentralised health and social care systems and other developed welfare states with a social health insurance system.

Our findings offer several implications for future research. Firstly, it would be important to expand this qualitative study to include more patient perspectives on barriers to obtain their views on how their needs and resources can be better supported. Secondly, future studies may investigate if and how ongoing reforms in health and social care systems (e.g., primary care units) address access barriers for people with chronic conditions identified by our study. Finally, further research will be helpful in conceptualising access along the different stages of a patient’s illness within a framework that is applied not only at the end of the care process but throughout. There is currently a gap in the evidence that would capture not only structural barriers to accessing health care but underpin indicators that are specific to the experience of people managing chronic conditions. This study contributes to the evidence by providing in-depth qualitative research – considering provider, expert, and researcher perspectives – on how patients with chronic conditions access care, including their barriers, and possible areas for improvement in health services to respond appropriately to patients’ specific needs.

## Conclusion

Barriers to healthcare access particularly concern patients with chronic conditions, resulting in an urgent need to improve health services according to patients’ specific needs. Based on our findings, barriers are strongly interlinked. On the supply side, patterns of poor patient-provider communication, lack of a holistic therapeutic approach, an urban-rural divide, strict separation between social care and the healthcare system and limited consultation time were among the barriers identified. On the demand side, patients’ ability to perceive a need and to subsequently seek and reach healthcare services was an important barrier, closely linked to a patient’s socio-economic status, health literacy and ability to pay. For health services to be properly accessible to patients with chronic conditions, timely and integrated care independent of social and economic resources, continuity of care, and significant improvements in patient-centred communication and coordination of care are paramount. Further, shortcomings in coordination, cooperation, and communication at the very beginning of patients’ treatment pathways are likely to impede future courses of treatment, leading to an exacerbation of existing barriers. The close interactions between poor communication, uncoordinated care pathways, and, ultimately, poorer health outcomes become particularly visible when addressing chronic conditions and confirm the importance of a holistic and patient-centred approach.

## Supplementary Information

**Additional file 1: Supplementary table 1.** Interview structure.

## Data Availability

The datasets generated and/or analysed during the current study are not publicly available due confidentiality obligations but are available from the corresponding author on reasonable request.

## Abbreviations

ATHIS: Austrian Health Interview Survey
GP: General practitioner
LBP: Low back pain
SHI: Social health insurance
VHI: Voluntary health insurance
