# Supplementary material for: Barriers to accessing health care for people with chronic conditions: a qualitative interview study
Source: BMC Health Serv Res. 2022 Aug 14;22:1037. doi: 10.1186/s12913-022-08426-z (PMC9375930; doi:10.1186/s12913-022-08426-z)
Supplement: Supplementary file 1 — Additional file 1: Supplementary table 1. Interview structure. [file 12913_2022_8426_MOESM1_ESM.docx]

# Supplementary material

*Supplementary table 1: Interview structure*

| **Focus** | **Key question** | **Follow up questions** |
| --- | --- | --- |
| Patient group |  | How would you describe the group of people just described, based on your knowledge, your experience? What are the first things that come to your mind? |
| Barriers | After introducing the model of Levesque et al.:  From your point of view, where do barriers exist in the context of health care for children with bronchial asthma/ working people with non-specific chronic low back pain (LBP) / older people with pre-existing mental illness (excluding dementia) living independently? | - Where do you see barriers specifically in the area you work in? - Can you think of barriers that are particularly common in a specific area of care (e.g. outpatient practices)? - Can you think of barriers particularly relevant for individuals with certain symptoms, medical conditions, diagnoses? - Can you imagine that barriers exist also outside your area of work/perception/activity? What could these be? |
| Influencing factors | From your point of view, what are possible reasons/ causes for barriers you have mentioned? | - Are there specific subgroups of children with bronchial asthma/ working people with non-specific chronic low back pain (LBP) / older people with pre-existing mental illness (excluding dementia) living independently that are particularly affected by these barriers? If so, why? - Why do you think barriers occur particularly often with this group? - Are the reasons related rather to the design of the system, or do barriers occur because of certain characteristics of these patients? |
| Recommendations | What would need to happen to reduce, eliminate, or make it easier to overcome the barriers you mentioned? | - Who could contribute to this? - Where do you see opportunities specifically in the area in which you work? - Can you think of measures, recommendations related to selected symptoms, clinical pictures, diagnoses? |
